# Risk and predictors of psoriasis in patients with breast cancer: a Swedish population-based cohort study

**DOI:** 10.1186/s12916-017-0915-4

**Published:** 2017-08-11

**Authors:** Haomin Yang, Judith S. Brand, Jingmei Li, Jonas F. Ludvigsson, Emilio Ugalde-Morales, Flaminia Chiesa, Per Hall, Kamila Czene

**Affiliations:** 1 Department of Medical Epidemiology and Biostatistics, Karolinska Institutet, Nobels Väg 12A, 171 77 Stockholm, Sweden; 2 Department of Pediatrics, Örebro University Hospital, Örebro, Sweden

## Abstract

**Background:**

The risk of psoriasis in patients with breast cancer is largely unknown, as available evidence is limited to case findings. We systematically examined the incidence and risk factors of psoriasis in patients with breast cancer.

**Methods:**

A Swedish nationwide cohort of 56,235 breast cancer patients (2001–2012) was compared to 280,854 matched reference individuals from the general population to estimate the incidence and hazard ratio (HR) of new-onset psoriasis. We also calculated HRs for psoriasis according to treatment, genetic, and lifestyle factors in a regional cohort of 8987 patients.

**Results:**

In the nationwide cohort, 599 patients with breast cancer were diagnosed with psoriasis during a median follow-up of 5.1 years compared to 2795 cases in the matched reference individuals. This corresponded to an incidence rate of 1.9/1000 person-years in breast cancer patients vs. 1.7/1000 person-years in matched reference individuals. Breast cancer patients were at an increased risk of psoriasis (HR = 1.17; 95% confidence interval (CI) = 1.07–1.28), especially its most common subtype (psoriasis vulgaris; HR = 1.33; 95% CI = 1.17–1.52). The risk of psoriasis vulgaris was highest shortly after diagnosis but remained increased up to 12 years. Treatment-specific analyses indicated a higher risk of psoriasis in patients treated with radiotherapy (HR = 2.44; 95% CI = 1.44–4.12) and mastectomy (HR = 1.54, 95% CI = 1.03–2.31). Apart from treatment-specific effects, we identified genetic predisposition, obesity, and smoking as independent risk factors for psoriasis in breast cancer patients.

**Conclusions:**

The incidence of psoriasis is slightly elevated among patients with breast cancer, with treatment, lifestyle, and genetic factors defining the individual risk profile.

**Electronic supplementary material:**

The online version of this article (doi:10.1186/s12916-017-0915-4) contains supplementary material, which is available to authorized users.

## Background

Psoriasis is a complex autoimmune skin disorder characterized by patches of abnormal skin. The prevalence of the condition has been estimated to be between 2% to 3% in the general population of Western countries [1, 2]. Common symptoms include red, inflamed skin and scaly plaques. More severe complications, such as inflammatory arthritis, can result in joint deformations and disability. Patients suffering from psoriasis typically report a poor health-related quality of life and endure significant social stigma [3–5].

Clinical observations suggest a potential increased risk of psoriasis in patients with breast cancer, which has mainly been attributed to skin trauma due to surgery [6] or radiotherapy-induced skin reactions [7]. Estimates of the incidence and relative risk of psoriasis as compared to the general population, however, are unknown, as the available evidence is limited to case reports with a predominant focus on patients who were previously diagnosed with psoriasis [8–11]. Moreover, no study to date has systematically examined the impact of different treatment-related factors including surgery and radiotherapy on psoriasis incidence, despite the fact that skin trauma has been shown to be a triggering factor for more than half of the new-onset psoriasis cases [12]. Considering the rising incidence of breast cancer and the large proportion of patients undergoing radiotherapy and surgery, it is of importance to better understand the risk of psoriasis in women treated with such modalities.

Psoriasis is an autoimmune disease with genetic variants and lifestyle factors (including alcohol consumption, smoking, and obesity) influencing disease susceptibility [13–16]. Since cancer cells and immunosuppressive cancer treatments including chemotherapy can influence the immune system of patients with breast cancer, the contribution of genetic and lifestyle factors to psoriasis risk may be different in this patient population [17]. Whether risk factors identified in the general population predispose to psoriasis in these patients, however, has not been assessed previously.

We aimed to assess the relative risk and incidence rates of psoriasis in patients with breast cancer as compared to the general population, overall and by time since diagnosis. We specifically evaluated risks by radiotherapy and surgery as sources of skin trauma-triggering events. We also studied the impact of previously identified psoriasis risk factors (i.e., genetic predisposition and lifestyle factors) to comprehensively understand the factors influencing disease susceptibility in this patient population.

## Methods

### Study populations

We analyzed two population-based cohorts: a Swedish nationwide cohort of breast cancer patients and a regional breast cancer cohort (Table 1, Additional file 1: Figure S1).

**Table 1:** Descriptive characteristics of the nationwide and regional breast cancer cohorts

	Nationwide cohort *n* = 56,235	Regional cohort *n* =8987
Cohort period	2001–2012	2001–2013
Age at diagnosis (years)		
Mean (SD)	60.1 (11.0)	58.6 (11.2)
Minimum–maximum	20–80	23–80
Duration of follow-up (years)		
Median (IQR)	5.1 (5.4)	7.7 (4.3)
Total no. of person-years at risk	307,684	68,243
Cases of psoriasis	599	150
Age at psoriasis diagnosis (SD)	62.5 (10.0)	63.6 (10.2)

*SD* standard deviation, *IQR* interquartile range

The nationwide cohort includes women diagnosed with primary invasive breast cancer at age 20–80 years between 2001 and 2011. In this cohort, follow-up is complete until 31 December 2012. The regional cohort includes women diagnosed with primary invasive breast cancer at age 20–80 years between 2001 and 2008; all patients in this cohort have complete follow-up until 31 December 2013

The nationwide cohort of breast cancer patients comprised women who were part of the 1990 national census of Sweden and were diagnosed with primary invasive breast cancer between 2001 and 2011, at age 20–80 years (*n* = 56,976). Information on breast cancer diagnoses was obtained through the Swedish Cancer Register, which was founded in 1958 and is managed by the National Board of Health and Welfare. Since the focus of our study is on the risk of new-onset psoriasis, patients who had been diagnosed with psoriasis before the date of the breast cancer diagnosis were excluded, leaving 56,235 patients in the cohort (Table 1). To compare the risk of psoriasis, we randomly sampled up to 5 women from the general female population matched on age, county of residence, and social economic status (obtained from the 1990 national census of Sweden, categorized as blue collar workers, white collar workers, self-employed workers, farmers, and others). Each reference individual was alive and free of cancer and psoriasis on the date of the matched patient’s diagnosis (the index date). In total, 321 women could not be matched to an index case, resulting in 280,854 matched reference individuals. Follow-up of the cohorts started from the index date (i.e., diagnosis date for breast cancer patients) and ended on 31 December 2012, date of death, date of emigration, date of a secondary cancer diagnosis, or date of psoriasis diagnosis, whichever came first. Information on death and emigration was obtained through cross-linking the cohorts to the Swedish Causes of Death Register and the Swedish Migration Register, using the unique personal identity number.

LIBRO-1 is a regional breast cancer cohort including women diagnosed with primary invasive breast cancer (at age 20–80 years) between 2001 and 2008 in the Stockholm-Gotland area (*n* = 8987). Detailed information on tumor characteristics and treatment at baseline was available in LIBRO-1 through linkage to the Swedish breast cancer quality registers (Information Networks for Cancer Treatment and Regional Cancer Center Stockholm-Gotland), including tumor size, estrogen receptor status, metastasis status, as well as information on intended treatment in terms of surgery, radiotherapy, chemotherapy, and endocrine therapy. The LIBRO-1 cohort was linked to the Swedish Causes of Death Register and the Swedish Migration Register to obtain information on date of death and emigration. Person-time was defined in the same way as for the nationwide cohort, except for an extension of the follow-up until 31 December 2013.

### Psoriasis diagnoses

All psoriasis cases were identified through the Swedish Patient Register [18], which was established in 1964 and achieved nationwide coverage for inpatient hospitalizations in Sweden since 1987. From 2001, hospital-based outpatient physician visits have also been reported by Swedish counties. Diagnoses were coded according to the 7^th^–10^th^ Swedish Revision of the International Classification of Diseases (ICD) codes (ICD-7 (1964–1968): 706; ICD-8 and ICD-9 (1969–1996): 696; ICD-10 (1997–present): L40). The validity of a psoriasis diagnosis in the inpatient and outpatient registers is 81% [19]. The first psoriasis subtype recorded was defined by the use of the ICD-10 code into: psoriasis vulgaris (L40.0), palmoplantar pustulosis (L40.3), and arthropathic psoriasis (L40.5). To ensure specificity, only primary discharge diagnoses were considered for the present analysis.

### Genetic and lifestyle risk factors

A subset of 4365 breast cancer patients within LIBRO-1 who were alive in 2009 consented to participate in a questionnaire-based study and gave a blood sample for genetic analyses. Patients’ lifestyle information on cigarette smoking, body mass index (BMI), alcohol consumption, and physical activity prior to diagnosis was retrieved from the self-reported questionnaire. Genotyping was carried out using a custom Illumina iSelect array (iCOGS) comprising 211,155 single nucleotide polymorphisms (SNPs) [20]. Details of the iCOGS array design, sample handling, and quality control processes have been described elsewhere [20]. To assess genetic predisposition to psoriasis, we selected 35 genome-wide significant SNPs reported by a recent meta-analysis of psoriasis genome-wide association studies (GWASs) [21] for constructing a polygenic risk score (PRS) using a scoring routine in the PLINK software v1.9 [22]. All SNPs were not directly genotyped on iCOGS, but imputed instead using the 1000 Genomes Project March 2012 release as a reference [23]. All SNPs passed quality control (minor allele frequency (MAF) ≥ 0.01 and R^2^/IMPUTE info-score ≥ 0.5), and for each individual breast cancer patient, a weighted PRS was calculated using the following formula:

$$ \mathrm{P}\mathrm{R}\mathrm{S}={\beta}_1{x}_1+{\beta}_2{x}_2+....{\beta}_k{x}_k+{\beta}_n{x}_n $$

where *β* is the per-allele log odds ratio (OR) for psoriasis associated with the risk allele for SNP _*k*_, *x*_*k*_ is the number of alleles for the same SNP (0, 1, 2), and *n* is the total number of disease SNPs included in the profile. The SNPs and corresponding ORs (weights) used for the derivation of the PRS are summarized in Additional file 1: Table S1. For analysis, patients were categorized by tertiles of the PRS.

### Statistical analysis

To assess the risk of psoriasis in patients with breast cancer, we used stratified Cox regression models (i.e., Cox regression models conditioned on the matching variables age, county of residence, and social economic status) and calculated hazard ratios (HRs) for psoriasis in patients with breast cancer, overall, and by dividing the follow-up time into periods of 0 to < 0.5 year, 0.5 to < 1 year, 1–5 years, and >5 years, and by age at diagnosis (20–44 years, 45–54 years, 55–64 years, and 65–80 years). The underlying time scale for all the analyses was time since diagnosis. The stratified Cox model has been a recommended analysis approach for matched cohort data, and it deals with the presence of potential confounders (measured and unmeasured) as well as any imbalances in the matching scheme caused by censoring [24–26]. Potential effect modification of age was tested by adding an interaction term of breast cancer (yes vs. no) with age (20–44 years, 45–54 years, 55–64 years, and 65–80 years) to the Cox model. We used Kaplan-Meier analyses to assess the cumulative incidence of psoriasis in breast cancer patients and matched reference individuals. This analysis approach does not account for the competing risk of death. Therefore, to address this potential bias, we also analyzed the data using competing risk models, treating mortality as a competing event.

To identify the risk factors for psoriasis in patients with breast cancer, we analyzed associations with cancer treatment in the regional cohort of breast cancer patients. The basic model (Model 1) was adjusted for age at breast cancer diagnosis and calendar period. In the multivariable model (Model 2), all treatment variables were entered for mutual adjustment as fixed covariates. To investigate potential confounding by disease severity, a sensitivity analysis was conducted in which tumor characteristics were added to Model 2. We further studied the effect of genetic predisposition and lifestyle factors in the subcohort with questionnaire and genotyping data. These analyses were conducted in the same manner as the analysis of cancer treatments, with a basic model (Model 1) adjusting for age at breast cancer diagnosis and calendar period, and a multivariable model (Model 2) including all risk factors, including identified treatment-related risk factors. Ordinary Cox regression was used for all risk factor analyses, and the proportional hazards assumption was tested using Schoenfeld residuals.

Statistical analyses were performed using SAS (version 9.4; SAS Institute Inc., Cary, NC, USA) and Stata software (version 14.0; Stata Corporation, College Station, TX, USA). The study was approved by the Regional Ethical Review Board in Stockholm.

## Results

### Risk of psoriasis in patients with breast cancer as compared to matched reference individuals

**Fig. 1:**
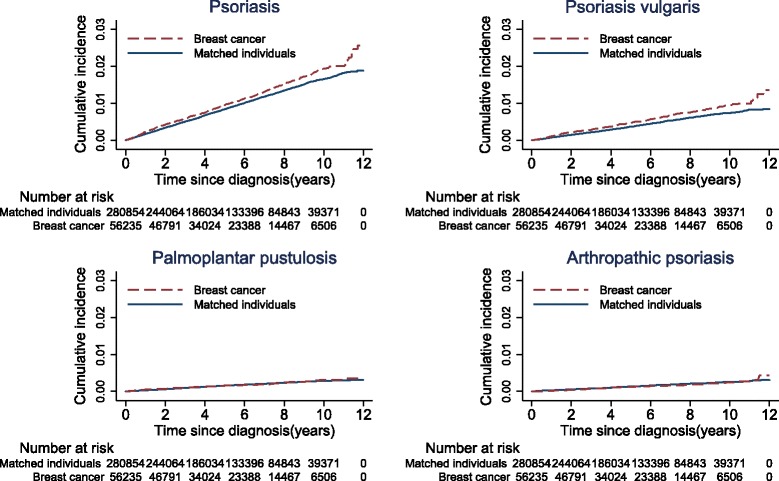
Cumulative incidence of psoriasis in the nationwide cohort of breast cancer patients and matched individuals. Kaplan-Meier estimates of the cumulative risk of psoriasis by time since diagnosis, in breast cancer patients and matched individuals from the general population

In total, 599 cases of psoriasis were observed during a median follow-up of 5.1 years in the nationwide breast cancer cohort compared to 2795 cases in the matched reference individuals. This corresponded to an incidence rate of 1.9/1000 person-years in breast cancer patients vs. 1.7/1000 person-years in the matched reference individuals, indicating a 0.2/1000 person-years of absolute risk increase (95% confidence interval (CI) = 0.1/1000–0.4/1000). The most common psoriasis subtype was psoriasis vulgaris (298 out of 599), followed by palmoplantar pustulosis and arthropathic psoriasis. The 5-year cumulative incidence of psoriasis in breast cancer patients and in the matched reference individuals was 1.0% and 0.8%, respectively (Fig. 1). These estimates were very similar to those observed in competing risk analyses, indicating that bias due to the competing risk of death is negligible.

Patients with breast cancer experienced a 17% increased risk of being diagnosed with psoriasis during follow-up (HR = 1.17; 95% CI = 1.07–1.28) (Table 2). The increased risk of psoriasis was mainly attributed to psoriasis vulgaris (HR = 1.33, 95% CI = 1.17–1.52), with no overall risk increase being observed for the other psoriasis subtypes. Analyses by time since diagnosis showed that the risk of psoriasis was highest in the second half of the first year after diagnosis (HR = 1.68, 95% CI = 1.30–2.19). The risk of psoriasis vulgaris was long-term increased, with the HR remaining significant between 5 and 12 years after diagnosis (HR = 1.33, 95% CI = 1.06–1.68). Risk of psoriasis did not vary among different age groups, and the interaction between a breast cancer diagnosis and age was not significant (*P* for interaction = 0.84).

**Table 2:** Hazard ratios for psoriasis in the nationwide breast cancer cohort

	No. of PYs	Any psoriasis		Psoriasis vulgaris		Palmoplantar pustulosis		Arthropathic psoriasis	
		Total/case no.	HR (95% CI)	Case no.	HR (95% CI)	Case no.	HR (95% CI)	Case no.	HR (95% CI)
Overall									
Matched reference cohort	1,666,038	280,854/2795	1.00 (Ref.)	1234	1.00 (Ref.)	488	1.00 (Ref.)	433	1.00 (Ref.)
Breast cancer cohort	307,684.8	56,235/599	**1.17 (1.07–1.28)**	298	**1.33 (1.17–1.52)**	95	1.04 (0.84–1.30)	75	0.94 (0.73–1.20)
Time since diagnosis									
**0 to < 0.5 year**									
Matched reference cohort	139,886.5	280,854/238	1.00 (Ref.)	86	1.00 (Ref.)	48	1.00 (Ref.)	51	1.00 (Ref.)
Breast cancer cohort	27,780.35	56,235/50	1.08 (0.80–1.47)	23	1.35 (0.85–2.14)	13	1.40 (0.76–2.59)	4	0.41 (0.15–1.13)
**0.5 to < 1 year**									
Matched reference cohort	138,587.1	278,473/228	1.00 (Ref.)	113	1.00 (Ref.)	27	1.00 (Ref.)	32	1.00 (Ref.)
Breast cancer cohort	27,301.1	55,033/75	**1.68 (1.30–2.19)**	37	**1.66 (1.15–2.41)**	15	**2.88 (1.52–5.43)**	5	0.80 (0.31–2.07)
**1–5 years**									
Matched reference cohort	861,202.7	275,870/1451	1.00 (Ref.)	629	1.00 (Ref.)	281	1.00 (Ref.)	219	1.00 (Ref.)
Breast cancer cohort	162,011.6	54,156/296	1.09 (0.96–1.24)	146	**1.27 (1.06–1.52)**	43	0.81 (0.58–1.12)	44	1.07 (0.77–1.48)
**> 5 years**									
Matched reference cohort	526,362	159,147/878	1.00 (Ref.)	406	1.00 (Ref.)	132	1.00 (Ref.)	131	1.00 (Ref.)
Breast cancer cohort	90,591.81	28,430/178	1.17 (0.99–1.38)	92	**1.33 (1.06–1.68)**	24	1.02 (0.66–1.60)	22	0.98 (0.62–1.56)
Age at breast cancer diagnosis									
**20–44 years**									
Matched reference cohort	158,527.2	25,361/185	1.00 (Ref.)	75	1.00 (Ref.)	30	1.00 (Ref.)	46	1.00 (Ref.)
Breast cancer cohort	28,908.36	5099/43	1.35 (0.97–1.90)	24	**1.88 (1.17–3.00)**	6	1.08 (0.45–2.62)	6	0.82 (0.35–1.94)
**45–54 years**									
Matched reference cohort	389,187.5	61,404/707	1.00 (Ref.)	285	1.00 (Ref.)	155	1.00 (Ref.)	122	1.00 (Ref.)
Breast cancer cohort	72,753.48	12,289/151	1.16 (0.97–1.38)	72	**1.39 (1.07–1.81)**	28	0.95 (0.63–1.43)	22	0.97 (0.61–1.53)
**55–64 years**									
Matched reference cohort	549,425.4	89,347/1067	1.00 (Ref.)	447	1.00 (Ref.)	213	1.00 (Ref.)	172	1.00 (Ref.)
Breast cancer cohort	102,753.2	17,850/229	1.14 (0.99–1.32)	109	**1.27 (1.03–1.58)**	42	1.04 (0.74–1.45)	23	0.74 (0.47–1.15)
**65–80 years**									
Matched reference cohort	568,898.3	104,742/836	1.00 (Ref.)	427	1.00 (Ref.)	90	1.00 (Ref.)	93	1.00 (Ref.)
Breast cancer cohort	103,269.8	20,997/176	1.16 (0.99–1.38)	93	**1.27 (1.01–1.59)**	19	1.21 (0.73–2.01)	24	1.30 (0.82–2.07)

*CI* confidence interval, *no.* number, *PYs* person-years, *HR* hazard ratio

Hazard ratio of psoriasis in the nationwide breast cancer cohort compared to age, residence place, and social economic status matched Swedish female population (age 20–80). Significant associations are denoted in boldface. *P* values for the test of interaction term of breast cancer diagnosis and age groups are 0.84, 0.49, 0.91, and 0.37, respectively, for psoriasis overall, psoriasis vulgaris, palmoplantar pustulosis, and arthropathic psoriasis

### Risk of psoriasis by treatment, genetic predisposition, and lifestyle factors in patients with breast cancer

Analyses by treatment characteristics showed no effect of chemotherapy and endocrine therapy on psoriasis risk (Table 3). Radiotherapy, in contrast, was associated with a twofold increased risk after adjusting for surgery, chemotherapy, and endocrine therapy (HR = 2.44; 95% CI = 1.44–4.12). An increased risk of psoriasis was also observed in breast cancer patients who underwent mastectomy, compared to those who had a lumpectomy (HR = 1.54, 95% CI = 1.03–2.31) in the multivariable adjusted model (Model 2). A sensitivity analysis with additional adjustment for tumor characteristics yielded similar results (Additional file 1: Table S2).

**Table 3:** Hazard ratios for psoriasis in the regional breast cancer cohort according to treatment characteristics

	Total no.	No. of cases	HR (95% CI)	
			Model 1	Model 2
Endocrine therapy				
No	1533	27	1.00 (Ref.)	1.00 (Ref.)
Yes	7100	121	0.90 (0.59–1.36)	0.80 (0.52–1.24)
Chemotherapy				
No	5544	102	1.00 (Ref.)	1.00 (Ref.)
Yes	3070	46	0.82 (0.57–1.19)	0.70 (0.47–1.04)
Radiotherapy				
No	2061	23	1.00 (Ref.)	1.00 (Ref.)
Yes	6574	125	**1.78 (1.14–2.78)**	**2.44 (1.44–4.12)**
Surgery				
Lumpectomy	5203	94	1.00 (Ref.)	1.00 (Ref.)
Mastectomy	3459	55	0.96 (0.69–1.34)	**1.54 (1.03–2.31)**

*CI* confidence interval, *Total no.* number of breast cancer patients, *No. of cases* number of psoriasis cases, *HR* hazard ratio

Model 1: adjusted for age and calendar period of breast cancer diagnosis. Model 2: Model 1 plus all the treatment factors. Significant associations are denoted in boldface. Missingness on all variables is <5%. No evidence of non-proportional hazards was found

Table 4 shows the association of genetic and lifestyle factors with psoriasis risk in patients with breast cancer. A significantly increased risk of psoriasis was found among patients in the highest tertile of the psoriasis PRS compared to those having lower genetic scores (HR = 2.94; 95% CI = 1.57–5.49, *P* for trend < 0.001). In the multivariable model (Model 2), regular cigarette smoking for more than 1 year prior to diagnosis (HR = 1.59; 95% CI = 1.00–2.52) and a BMI larger than 30 kg/m^2^ at diagnosis (HR = 2.10; 95% CI = 1.20–3.68) significantly increased the risk of psoriasis in breast cancer patients. In the multivariable model, a high level of physical activity (more than 2 h per week) prior to diagnosis was shown to protect breast cancer patients from psoriasis; however, this effect was not significant (HR = 0.59; 95% CI = 0.33–1.03).

**Table 4:** Hazard ratios for psoriasis in the regional breast cancer cohort according to genetic and lifestyle factors

	Total no.	No. of cases	HR (95% CI)	
			Model 1	Model 2
PRS score				
Tertile 1	1440	13	1.00 (Ref.)	1.00 (Ref.)
Tertile 2	1442	36	**2.74 (1.45–5.17)**	**2.83 (1.50–5.34)**
Tertile 3	1483	40	2.94 (1.57–5.49)*	2.98 (1.59–5.58)*
BMI				
< 25 kg/m^2^	2331	40	1.00 (Ref.)	1.00 (Ref.)
25–30 kg/m^2^	1434	28	1.18 (0.73–1.92)	1.15 (0.71–1.87)
> 30 kg/m^2^	536	19	**2.29 (1.32–3.98)**	**2.10 (1.20–3.68)**
Physical activity per week				
0 h	762	21	1.00 (Ref.)	1.00 (Ref.)
0–2 h	1645	36	0.77 (0.45–1.33)	0.77 (0.44–1.32)
> 2 h	1910	30	**0.56 (0.32–0.98)**	0.59 (0.33–1.03)
Regular smoker (cigarette smoking >1 year)				
No	1773	26	1.00 (Ref.)	1.00 (Ref.)
Yes	2546	62	**1.65 (1.04–2.61)**	**1.59 (1.00–2.52)**
Alcohol consumption				
No	104	2	1.00 (Ref.)	1.00 (Ref.)
Yes	2861	58	1.03 (0.25–4.22)	1.12 (0.27–4.70)

*Total no.* number of breast cancer patients, *No. of cases* number of psoriasis cases, *HR* hazard ratio, *CI* confidence interval, *BMI* body mass index, *PRS* polygenic risk score

Analyses were based on a subset of the regional cohort with information on genetic and lifestyle factors. Significant associations are denoted in boldface. Genetic predisposition for psoriasis was defined by a PRS including 35 genetic variants for psoriasis susceptibility. Patients were grouped into tertiles by their genetic risk. Model 1: adjusted for age and calendar period of breast cancer diagnosis. Model 2: all of the risk factors were put into the model, including radiotherapy and mastectomy. Missingness on all variables is <5%, except for alcohol consumption (32.1%, *N* = 1400). No evidence of non-proportional hazards was found

*P for trend < 0.001, tested by log-linear trend test

## Discussion

### Key results

The incidence of psoriasis was 17% higher in breast cancer patients compared to the matched reference individuals in this study. The relative risk of psoriasis was seen within the first year after diagnosis and was mainly attributed to an increased risk of psoriasis vulgaris (33% higher). Treatment-specific analyses indicated an increased risk of psoriasis in patients treated with radiotherapy and mastectomy. Apart from treatment-specific effects, we identified genetic predisposition, obesity, and smoking as independent risk factors for psoriasis in patients with breast cancer.

### Interpretation

Previous studies have reported a higher risk of skin disorders such as dermatitis and skin infection in patients with breast cancer [27, 28]. We found a slightly increased risk of psoriasis in breast cancer patients as compared to the general population. However, the overall incidence of psoriasis is low, and the absolute risk differences observed were also rather small. Of note, the cumulative incidence estimates of the current study are based on primary clinical diagnoses of psoriasis from the patient register (1%) and are lower than the estimate reported in a previous study (13%) using skin screening results [7]. The use of a clinical definition may have resulted in underestimation of the incidence estimates while capturing the more severe cases.

The increased risk of psoriasis after breast cancer is clinically plausible, as some treatments for breast cancer can cause dermatological side effects. In the regional cohort, we found an association between radiotherapy and risk of psoriasis. Skin trauma (a burn, scratch, bruise, cut, etc.) has been reported as a triggering factor for 43–76% of incident psoriasis cases [12]. The highest risk of psoriasis within the first year after diagnosis could also partly be explained by trauma caused by ionizing radiation. Compared to lumpectomy, we observed a significantly higher risk of psoriasis in breast cancer patients with mastectomy in the multivariable model. Patients with mastectomy usually experience more wound complications and delayed wound healing [29]. As the two main pathogenic features of psoriasis (abnormal keratinocyte differentiation and hyperproliferation of keratinocytes) are also secondary to the altered development of the normal wound healing process [30], prolonged wound healing could trigger psoriasis onset. Additionally, an increased risk of psoriasis could be explained by psychological reactions to the disease diagnosis and treatment decision, since a severe stressful life event has been identified as a potential trigger for psoriasis [31]. Both factors (treatment and stress reaction to the actual diagnosis) could potentially explain the observed peak in psoriasis incidence shortly after diagnosis.

Our study further identified obesity, smoking, and a high genetic predisposition as independent risk factors of psoriasis in patients with breast cancer. Psoriasis is a highly heritable disease; the heritability rate is estimated to be 70% [13]. Previous studies have reported substantially higher relative risks of psoriasis when comparing the highest to the lowest quartile of the PRS, with risks being inversely correlated to the age of psoriasis diagnosis [17, 32, 33]. Although these results are not directly comparable to our findings, lower relative risks with genetic predisposition are anticipated in our study population because of the older age at psoriasis onset (after diagnosis of breast cancer). Smoking and increased BMI are also established risk factors for psoriasis in the general population [34]. The effect of smoking and obesity in the breast cancer cohort was comparable to effects previously observed in the general population [14, 16].

The main strength of our study is the large sample size and the population-based design using Swedish health registers, which minimizes selection and information biases. Other strengths include the abundant information of treatment, lifestyle, and genetic information in the regional breast cancer cohort. We also acknowledge several limitations. The validity (positive predictive value) of psoriasis diagnoses in the Swedish Patient Register is about 81% [19], which indicates a possibility of misclassification (e.g., misclassified radio dermatitis as psoriasis). Although mild cases of radio dermatitis and psoriasis have some symptoms in common (erythema on the skin and sometimes desquamation), severe psoriasis is quite different from radio dermatitis [35], and in contrast to dermatitis, does not disappear after a couple of weeks. As the patient register mostly includes the severe cases of psoriasis, this misclassification should not have influenced our results. Furthermore, because of increased medical surveillance, breast cancer patients may have received a diagnosis that would, in a healthy person, continue to be undiagnosed. However, the long-term risk of psoriasis vulgaris (up to 12 years) argues against surveillance bias being a pure explanation for the increased psoriasis risk observed.

## Conclusions

The overall risk of psoriasis is slightly increased among patients with breast cancer compared to the general population. While the relative risk of psoriasis is highest within the first year of diagnosis, the risk of psoriasis vulgaris remained increased up to 12 years. Independent risk factors of psoriasis in breast cancer patients are radiotherapy, mastectomy, smoking, obesity, and a high genetic predisposition. Our findings emphasize the complex etiology of psoriasis in patients with breast cancer.

## Additional file

Additional file 1: Table S1. List of single nucleotide polymorphisms (SNPs) used for constructing the polygenic risk score (PRS) for psoriasis. **Table S2.** Hazard ratios (HRs) for psoriasis in the regional breast cancer cohort according to treatment and adjusted for tumor characteristics. **Figure S1.** Flow diagram of analytic cohort. (DOCX 53 kb)

## Abbreviations

ICD: International Classification of Diseases
CI: Confidence interval
HR: Hazard ratio
PRS: Polygenic risk score
SNP: Single nucleotide polymorphism
BMI: Body mass index
GWAS: Genome-wide association study
MAF: Minor allele frequency
OR: Odds ratio.
