# Supplementary material for: Risk and predictors of psoriasis in patients with breast cancer: a Swedish population-based cohort study
Source: BMC Med. 2017 Aug 11;15:154. doi: 10.1186/s12916-017-0915-4 (PMC5553678; doi:10.1186/s12916-017-0915-4)
Supplement: Additional file 1: Table S1. — List of single nucleotide polymorphisms (SNPs) used for constructing the polygenic risk score (PRS) for psoriasis. Table S2. Hazard ratios (HRs) for psoriasis in the regional breast cancer cohort according to treatment and adjusted for tumor characteristics. Figure S1. Flow diagram of analytic cohort. (DOCX 53 kb) [file 12916_2017_915_MOESM1_ESM.docx]

**Additional file 1**

**Table S1.** List of single nucleotide polymorphisms (SNPs) used for constructing the polygenic risk score (PRS) for psoriasis.

**Table S2.** Hazard ratios (HRs) for psoriasis in the regional breast cancer cohort according to treatment and adjusted for tumor characteristics

**Figure S1.** Flow diagram of analytic cohort

**Table S1**. List of single nucleotide polymorphisms (SNPs) used for constructing the polygenic risk score (PRS) for psoriasis.

| Chr | SNP | Risk allele | OR | INFO | MAF |
| --- | --- | --- | --- | --- | --- |
| 1 | rs11121129 | A | 1.13 | 0.81 | 0.29 |
| 1 | rs6677595 | T | 1.26 | 0.68 | 0.36 |
| 1 | rs7536201 | C | 1.13 | 0.84 | 0.49 |
| 1 | rs7552167 | G | 1.21 | 1.00 | 0.14 |
| 1 | rs9988642 | T | 1.52 | 0.97 | 0.05 |
| 2 | rs10865331 | A | 1.12 | 0.70 | 0.35 |
| 2 | rs17716942 | T | 1.27 | 1.00 | 0.12 |
| 2 | rs62149416 | T | 1.17 | 0.99 | 0.37 |
| 5 | rs12188300 | T | 1.58 | 0.65 | 0.07 |
| 5 | rs1295685 | G | 1.18 | 1.00 | 0.22 |
| 5 | rs2233278 | C | 1.59 | 0.63 | 0.05 |
| 5 | rs27432 | A | 1.2 | 0.97 | 0.29 |
| 6 | rs2451258 | C | 1.12 | 0.86 | 0.31 |
| 6 | rs33980500 | T | 1.52 | 0.99 | 0.07 |
| 6 | rs582757 | C | 1.23 | 0.69 | 0.30 |
| 6 | rs9504361 | A | 1.12 | 1.00 | 0.45 |
| 7 | rs2700987 | A | 1.11 | 0.62 | 0.41 |
| 9 | rs10979182 | A | 1.12 | 1.00 | 0.42 |
| 9 | rs11795343 | T | 1.11 | 0.98 | 0.43 |
| 10 | rs1250546 | A | 1.1 | 0.55 | 0.42 |
| 11 | rs3802826 | A | 1.12 | 0.83 | 0.48 |
| 11 | rs4561177 | A | 1.14 | 0.50 | 0.42 |
| 11 | rs645078 | A | 1.09 | 0.99 | 0.41 |
| 12 | rs2066819 | C | 1.39 | 0.98 | 0.07 |
| 14 | rs8016947 | G | 1.16 | 0.95 | 0.44 |
| 16 | rs12445568 | C | 1.16 | 0.94 | 0.37 |
| 16 | rs367569 | C | 1.13 | 0.86 | 0.25 |
| 17 | rs11652075 | C | 1.11 | 0.89 | 0.49 |
| 17 | rs28998802 | A | 1.22 | 0.94 | 0.13 |
| 17 | rs963986 | C | 1.15 | 0.84 | 0.17 |
| 18 | rs545979 | T | 1.12 | 1.00 | 0.28 |
| 19 | rs34536443 | G | 1.88 | 0.50 | 0.03 |
| 19 | rs892085 | A | 1.17 | 0.63 | 0.47 |
| 20 | rs1056198 | C | 1.16 | 0.91 | 0.43 |
| 22 | rs4821124 | C | 1.13 | 0.96 | 0.22 |

Risk alleles and associated odds ratios (OR) which were used as weights for the construction of the PRS, as obtained from a meta-analysis of psoriasis GWAS (Tsoi *et al.*, 2012). INFO and MAF denote the quality of imputation and minor allele frequency in the LIBRO-1 dataset, respectively.

**Table S2**. Hazard ratios (HRs) for psoriasis in the regional breast cancer cohort according to treatment and adjusted for tumor characteristics

|  | 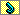**Total No.** | **No. of Cases** | **HR (95% CI)** |
| --- | --- | --- | --- |
| **Endocrine therapy** |  |  |  |
| No | 1,533 | 27 | 1.00 (REF) |
| Yes | 7,100 | 121 | 0.87 (0.44-1.71) |
| **Chemotherapy** |  |  |  |
| No | 5,544 | 102 | 1.00 (REF) |
| Yes | 3,070 | 46 | 0.70 (0.46-1.06) |
| **Radiotherapy** |  |  |  |
| No | 2,061 | 23 | 1.00 (REF) |
| Yes | 6,574 | 125 | **2.46 (1.45-4.18)** |
| **Surgery** |  |  |  |
| Lumpectomy | 5,203 | 94 | 1.00 (REF) |
| Mastectomy | 3,459 | 55 | **1.62 (1.05-2.49)** |

Abbreviations: CI = confidence interval. Total No.= Number of breast cancer patients. No. of Cases= Number of psoriasis cases. HR= Hazard ratio. The regression model adjusted for age and calendar period of breast cancer diagnosis, all the treatment factors, tumor size, estrogen receptor status, and metastasis status. Significant associations are denoted in bold. Missingness on all variables is < 5%. No evidence of non-proportional hazards was found.

**Figure S1.**

All invasive breast cancer patients diagnosed in Sweden between 2001 and 2011 *n*= 69,521

**Regional Breast Cancer cohort**

Patients living in the Stockholm /Gotland region during 2001-2008

*n* =10,007

**Corresponding General Population**

Women in Sweden during census 1990

*n*=4,343,853

**Nationwide Breast Cancer cohort**

All patients living in Sweden during 1990 census

*n*=67,209

Subcohort with genetic and lifestyle information (Follow up to Dec 31, 2013)

Age= 23-80

*n* =4,365

Women diagnosed with primary invasive breast cancer (Follow up to Dec 31, 2013)

Age= 23-80

*n* =8,987

Women diagnosed with primary invasive breast cancer (Follow up to Dec 31, 2012)

Age= 20-80

*n* =56,235

Matched cohort on age, county of residence and social economic status (Follow up to Dec 31, 2012)

Age= 20-80

*n*=280,854
